# Occupational health issues in marine and freshwater research

**DOI:** 10.1186/1745-6673-7-4

**Published:** 2012-03-19

**Authors:** Glenn Courtenay, Derek R Smith, William Gladstone

**Affiliations:** 1 School of Health Sciences, Faculty of Health, University of Newcastle, Central Coast Campus, Ourimbah, Australia; 2 School of the Environment, University of Technology, Sydney, Australia

**Keywords:** Exposure, Hazard, Injury, Occupational health, Research, Risk

## Abstract

Marine and freshwater scientists are potentially exposed to a wide variety of occupational hazards. Depending on the focus of their research, risks may include animal attacks, physiological stresses, exposure to toxins and carcinogens, and dangerous environmental conditions. Many of these hazards have been investigated amongst the general population in their recreational use of the environment; however, very few studies have specifically related potential hazards to occupational exposure. For example, while the incidence of shark and crocodile attacks may invoke strong emotions and the occupational risk of working with these animals is certainly real, many more people are stung by jellyfish or bitten by snakes or dogs each year. Furthermore, a large proportion of SCUBA-related injuries and deaths are incurred by novice or uncertified divers, rather than professional divers using aquatic environments. Nonetheless, marine and freshwater research remains a potentially risky occupation, and the likelihood of death, injury and long-term health impacts still needs to be seriously considered.

## Introduction

Although employment in the field of environmental research is often seen as glamorous, adventurous and exciting, as with many other occupations, specific hazards must be considered in order to maximise safety during routine performance of required tasks. Marine and freshwater scientists are potentially exposed to a wide variety of occupational hazards, and depending on the focus of their research, risks may include animal attacks, physiological stresses, exposure to toxins and carcinogens, and dangerous environmental conditions [1-4]. Furthermore, technological developments have expanded the range of environments and conditions in which marine and freshwater researchers are capable of working. For example, advances in dry suit technology have enabled broader polar research, modern ship design has allowed access to open ocean for longer periods of time, and developments in submarine technology has resulted in exploration of deeper water.

While procedures can be implemented to reduce the occupational risks associated with their field, environmental researchers must remain cautious of potential hazards. Marine and freshwater research remains a potentially risky occupation, and the likelihood of death, injury and long-term health impacts needs to be seriously considered. Although a substantial quantity of literature deals with various safety issues and the dangers from biota that this group may experience during their work [5,6], relatively few studies have focused on the specific occupational health aspects of this group, and few if any published studies describing occupational risk are research-specific. Consequently, much of this discussion explores the potential hazards that aquatic researchers may experience and attempts to relate these hazards to marine and freshwater research activity. Risks faced by SCUBA divers, for example, while being well-documented, have not generally been considered from an occupational perspective and much of the relevant literature pertains to the recreational diving community. In contrast, the consequences of increased exposure to specific hazards that marine and freshwater scientists may experience due to their activities, whether they be in the laboratory or the field, have received little attention, and as such, the magnitude of the problem is relatively unknown. For example, environmental scientists' potential exposure to toxic substances and infection is increasing [5,6] however, despite the relevance to occupational health, relatively little research has been conducted on the consequences of this increased exposure. Furthermore, there is the issue of risk perceptions versus *actual *(or quantifiable) risk in work-related hazard analysis [7,8]. Previous studies have suggested that inaccurate risk perceptions are most likely when unusual and dramatic injuries are sustained (especially animal-related) [9]; injuries which are clearly within the occupational health domain of marine and freshwater researchers (for example, shark attacks). As a result, the current paper reviews studies relating to issues which may impact on occupational health in marine and freshwater research.

## Materials and methods

An extensive literature review was conducted to examine the topic of occupational health in marine and freshwater field research. Only English-language reports were included. Initially, a list of potential risks, hazards and health issues were determined, classified and then a search of relevant headings such as 'animal attacks', 'SCUBA injuries', 'scientists occupational health' was conducted using Google Scholar. Marine and freshwater research, however, can occur on rock platforms, beaches, rivers, creeks, estuaries, coastal waters, subtidal (via scuba diving), open ocean, deep ocean (via submersibles) and polar areas. The field contexts can include: small boat and ship-based research, walking on beaches and rock platforms, wading through creeks, diving, snorkelling, and even may involve taking observations from a low flying helicopter. Therefore, while the focus of the present review was occupational health issues experienced by marine and freshwater researchers, many of the hazards and health issues identified are general in nature and are not necessarily specific to the aquatic researcher.

## Results

### Prevalence of occupational health problems

The literature pertaining to the risk of injuries in some situations which an environmental researcher might experience is extensive [1,10,11]. Depending on the type of research, occupational risks may include: exposure to broken glass and needle-stick injuries, boating incidents due to collisions with submerged objects or other boats, poor quality or lack of sufficient life jackets, injury due to lifting heavy objects and/or dropping them on one's toes, equipment failure due to poor maintenance, lack of sufficient work space on deck and the subsequent stress experienced by crew. Further risk may be due to exposure to environmental conditions such as: floods, landslides, bushfires, storms (and subsequent stormwater flow), lightning, tidal variation, waves, tsunamis or even skin cancer. Clearly, therefore, many of the aforementioned risks may also be applicable to the general population.

### Animal-related issues

A seemingly endless list of vertebrates and invertebrates are capable of causing injury or death to a marine or freshwater researcher (Table 1). However, despite the potential for exposure to these organisms, the likelihood of fatality to an aquatic researcher is low and the risk of injury can be minimised with sensible precautions. For example, in a 5-year study of injuries attributed to marine animals in Victoria, Australia [12], fishes (41%), stingrays (22%), jellyfish (21%), shellfish (8%), and sharks, crayfish and coral (2-3% each) were incriminated, particularly during the summer months. Most injuries were to the hands and resulted from spikes, spines or barbs; and there were no fatalities. While available data does not identify whether the victims were involved in marine research, less than 5% of these injuries occurred while the victim was in some form of employment.

**Table 1:** Animal-related occupational health issues in marine and freshwater research

Animal-type	Title of Article	Year^a^	Authors
Porifera	Clinical effects of stings by sponges of the genus Tedania and a review of sponge stings worldwide	2005	Isbister & Hooper [10]

Cnidaria	Prospective study of jellyfish stings from tropical Australia, including the major box jellyfish *Chironex fleckeri*	2001	O'Reilly et al. [13]

	Growth and age determination of the tropical Australian cubozoan *Chiropsalmus *sp.	2004	Gordon et al. [14]

	Prospective study of *Chironex fleckeri *and other box jellyfish stings in the "Top End" of Australia's Northern Territory	2005	Currie & Jacups [15]

	Australian venomous jellyfish, envenomation syndromes, toxins and therapy	2006	Tibballs [16]

	Inferring distributions of chirodropid box-jellyfishes (Cnidaria: Cubozoa) in geographic and ecological space using ecological niche modeling	2009	Bentlage et al. [17]

	Quantifying movement of the tropical Australian cubozoan *Chironex fleckeri *using acoustic telemetry	2009	Gordon & Seymour [18]

Molluscs	Blue-ringed octopus (Hapalochlaena sp.) envenomation of a 4-year-old boy: a case report	2008	Cavazzoni [19]

Fish	Catfish stings in Mississippi	1995	Das et al. [20]

	Stonefish envenomations of the hand - a local marine hazard: a series of 8 cases and review of the literature	2004	Lee et al. [21]

	Moray eel attack in the tropics: a case report and review of the literature	2004	Riordan et al. [22]

	Venomous fish injuries along the Israeli Mediterranean coast: scope and characterization	2008	Gweta et al. [23]

Sharks	Stingray injuries of the foot. Two case reports	1990	Burk & Richter [24]

	Infections after shark attacks in Australia	1997	Royle et al. [25]

	Threatened fishes of the world: *Glyphis gangeticus *(Müeller & Henle, 1839) (Carcharhinidae)	1997	Compagno [26]

	Pathologic features of fatal shark attacks	2000	Byard et al. [27]

	The cutaneous cellular infiltrate to stingray envenomization contains increased TIA + cells	2000	Germain et al. [28]

	The anatomy of a shark attack: a case report and review of the literature	2001	Caldicott et al. [29]

	Shark attack: Review of 86 consecutive cases	2001	Woolgar et al. [30]

	The hunting strategy of white sharks (*Carcharodon carcharias*) near a seal colony	2001	Klimley et al. [31]

	Pattern of stingray injuries reported to Texas poison centers from 1998 to 2004	2005	Forrester [32]

	White shark attacks upon humans in California and Oregon, 1993-2003	2006	McCosker & Lea [11]

	A review of shark agonistic displays: comparison of display features and implications for shark-human interactions	2007	Martin [33]

	Movement and distribution of young bull sharks *Carcharhinus leucas *in a variable estuarine environment	2008	Heupel & Simpfendorfer [34]

	Stingray poisoning, a careless aspect in México	2008	Rodríguez et al. [35]

	The evaluation, management, and prevention of stingray injuries in travelers	2008	Diaz [36]

	Shark attacks in Dakar and the Cap Vert Peninsula, Senegal: low incidence despite high occurrence of potentially dangerous species	2008	Trape [37]

	Shark attack outbreak off Recife, Pernambuco, Brazil: 1992-2006	2008	Hazin et al. [38]

	Finding of Inquest	2008	SACC [2]

	Sharks and Rays of Australia	2009	Last & Stevens [39]

	Movement patterns and water quality preferences of juvenile bull sharks (*Carcharhinus leucas*) in a Florida estuary	2009	Ortega et al. [40]

	Catching and tracking the world's largest Zambezi (bull) shark *Carcharhinas leucas *in the Breede Estuary, South Africa: the first 43 hours	2009	McCord & Lamberth [41]

	International Shark Attack File	2011	ISAF [42]

	SAS Peoples Shark Attack Related Incident File	2012	Shark Attack File.Info [43]

Reptiles	A Social History of the American Alligator	1991	Glasgow [44]

	Indication of crocodile recovery and management implications in crocodile conservation in Sabah	2004	Andau et al. [45]

	Crocodile attack in Australia: an analysis of its incidence and review of the pathology and management of crocodilian attacks in general	2005	Caldicott et al. [46]

	Alligator attacks on humans in the United States	2005	Langley [47]

	Two Sri Lankan cases of identified sea snake bites, without envenoming	2005	Senanayakea et al. [48]

	Alligator attacks in Southwest Florida	2006	Harding & Wolf [49]

	Review of the reintroduction programme of the Mugger crocodile *Crocodylus palustris *in Neyyar Reservoir, India	2006	Jayson et al. [50]

	Diet of the Nile crocodile (*Crocodylus niloticus*) in the Okavango Delta, Botswana	2008	Wallace & Leslie [51]

	Home range and movements of radio-tracked estuarine crocodiles (*Crocodylus porosus*) within a non-tidal waterhole	2008	Brien et al. [52]

	Size estimation, morphometrics, sex ratio, sexual size dimorphism, and biomass of Morelet's crocodile in northern Belize	2009	Platt et al. [53]

	Patterns of presentation of suspected snakebite in children in Western Australia from 1994 to 2004	2009	Rogers et al. [54]

Various animals	Safety of travel in South Africa: the Kruger National Park	2001	Durrheim et al. [9]

	An analysis of Marine Animal Injuries Presenting to Emergency Departments in Victoria, Australia	2002	Taylor et al. [12]

	Clinical toxinology - Where are we now?	2003	White et al. [55]

	Poisoning, envenomation, and trauma from marine creatures	2004	Perkins & Morgan [56]

	Rural/Farm Injury in Queensland. Queensland Injury Surveillance Unit	2006	QISU [57]

	Animal and human bite injuries in Victoria, 1998-2004	2007	MacBean et al. [58]

	Review article: Animal bites: an update for management with a focus on infections	2008	Dendle & Looke [59]

	Encounters with venomous sea-life	2011	Fernandez et al. [60]

^a ^Year of publication

Researchers are increasingly handling marine animals for tagging purposes and injuries may occur as a result of handling an animal after it has been caught. Additionally, stepping on a stingray, urchin or shell while conducting field work without protective footwear in shallows or along beaches may result in painful injury [12]. Stingrays were responsible for 30% of the 79 injuries to professional Israeli fishers by marine organisms during 2003-2004 and more than 100 bathers were stung at a Mazatlan beach area, Mexico, in 2008 [23,35]. Although they are usually a result of purely defensive gestures, around 1500 stingray injuries occur annually in the USA. However, since most stings result in only skin irritation or minor pain, the actual number of unreported cases is thought to be much higher [24,28,32,36].

Despite shark attacks being an emotive issue and generating much media attention and public apprehension, attacks are rare. Only about 50-80 unprovoked shark attacks on humans are confirmed annually worldwide and victims usually sustain only minor injuries [11,29,30,37,39,42]. A South African study of 86 victims of shark attack between 1980 and 1999 found that although 12% of the attacks were fatal, 80% of the survivors required only simple primary suture [30]. Since 2000, the fatality rate worldwide (7.0%) has been lower than previous decades, reflecting advances in beach safety practices and medical treatment (since death is usually due to lack of immediate resuscitation, haemorrhagic shock or drowning), and increased public awareness of avoiding potentially dangerous situations [27,29,42]. For example, in 2010, six fatalities resulting from unprovoked attack were recorded in South Africa (2), USA (2), Australia (1) and Egypt (1). Furthermore, in 2011, 117 shark attacks were reported worldwide, resulting in 17 fatalities including five in Australia and three in South Africa [43]. It is possible, however, that many attacks in developing countries are not reported and so precise figures may not be available [29,37].

The number of shark-human interactions occurring in a given year is related to the amount of time that humans spend in the sea and, with the design of modern wetsuits, individuals spend longer periods in the water. Surfers and body boarders were the group most affected in waters off Recife, Brazil between 1992 and 2006 when 47 incidents, including 17 fatalities, were reported [38]. Furthermore, the Woolgar et al. (2001) study found that surfers and board riders account for approximately 54% of victims in South Africa, snorkelers and spearfishers for 25%, swimmers 20% and scuba divers about 1%. Worldwide, the percentages are 51%, 8%, 41% and less than 1%, respectively [42], and relevantly, marine researchers who work in the sea are mostly scuba divers.

Victims most frequently suffer injuries to the lower limbs with most severe injuries involving one leg [27], and most non-serious injuries consist largely of puncture wounds and lacerations [30]. McCosker and Lea (2006) note that in 20 unprovoked attacks along the west coast of USA between 1993 and 2003, all victims, whether surfers, divers or swimmers, were wearing black or blue neoprene wetsuits, suggesting that the sharks mistook their victims for seals or sea-lions and that, consequently, the risk to wetsuit-clad marine researchers would be potentially greater. Relevantly, white sharks have been observed to spend almost 40% of each day patrolling within 1 km^2 ^of a colony of seals and sea lions on the California coast, and the duration and frequency of the sharks' visits to the waters off the pinniped colony deviated little during daytime, night time and twilight [31].

The danger to humans can be minimised, however, if the diver is able to recognise signs that a shark is becoming agitated and can respond appropriately [33]. Additionally, shark shield devices that can be worn by occupational and recreational divers are becoming more available. However, in 2002, a South Australian scallop diver who was incorrectly wearing such a device died as a result of an injury sustained in a shark attack [2]. Furthermore, in 2005, a South Australian research diver was fatally attacked by a shark while not wearing the supplied shark shield device due to concerns that the shields attracted sharks rather than deterred them [2].

Certain shark species are more frequently implicated in attacks. These include the great white shark, *Carcharodon carchararias*, the tiger shark, *Galeocerdo cuvier *and several members of the genus *Carcharhinus*, including the bull shark, *Carcharhinus leucas *[25,27,29,30,37,38]. Although bull sharks are also known to travel long distances into freshwater systems [34,40,41], little is known about the behaviour of genuine freshwater 'river' sharks such as *Glyphis *spp. which has been nominally implicated in attacks on humans in India. *Glyphis *spp., however, are critically endangered and contact with humans is rare [26].

Despite continued human population growth and increased interest in aquatic recreation, the number of shark attacks has generally levelled off since reaching a high of 80 in 2000 [42]. This is possibly due to worldwide over-fishing of sharks, better awareness of reducing shark-human interactions and improved efficiency in reporting and investigating attacks [29,42]. Furthermore, since bees, wasps and snakes are responsible for far more fatalities than sharks each year [29], compared to other causes of water-related deaths, the risk of shark attack appears negligible for the marine researcher not directly working with sharks [37].

Crocodilians are another group of animals which incite fear in humans. However, despite the encroachment of human and crocodilian populations on each other's territories, once again, attacks on humans are rare [46,49,59]. Although there has been a slight increase in non-fatal attacks in Australia since 1971, when saltwater crocodile numbers started increasing after protective legislation was introduced following 26 years of unregulated hunting, only 17 of 62 (27%) unprovoked crocodile attacks on humans between 1971 and 2004 resulted in fatality, and only three of the victims were researchers or wildlife rangers [45]. Similarly, thirty-five crocodile attacks recorded in Neyyar Wildlife Sanctuary, Kerala, India, since 1983 have resulted in only two fatalities [50]. Additionally, increasing crocodilian numbers in Sabah, Malaysia, and the USA have resulted in an increase in crocodile attacks and alligator-human interactions over a comparable period with 36% and 5% fatality rates, respectively [44,45,47]. Fatalities have also occurred in Africa where Nile crocodiles killed 23 people and injured 12 people in Namibia between 2000 and 2004, and killed 40 people and injured 8 people in Zambia between 2002 and 2004 [46]. However, frequency data is probably unreliable since attacks in remote areas tend not to be reported [46]. Furthermore, while numerous studies have been undertaken on crocodilian demographics [51-53], no data is available on the number of attacks that involved environmental researchers.

Most attacks occur during daylight when the victim is swimming or wading in relatively shallow water [46,47] with less than 10% of attacks occurring on victims in boats. However, canoes are more often attacked than other boats, suggesting that a canoe may resemble another crocodile or animal swimming in the water, when viewed from underwater [46]. Alternatively, it is possible that since there are more canoes than boats in developing countries, attacks on canoes would appear to be more frequent. Nevertheless, with sensible preparation, the potential hazard that crocodilians pose to the aquatic researcher is minimal.

In contrast with the small number of human interactions with sharks and crocodiles, millions of humans are affected by jellyfish annually resulting in approximately 15 deaths each year [55]. Although not true jellyfish, the 'Portuguese Man-O'-War', *Physalia physalis*, and the 'bluebottle', *Physalia utriculus*, are estimated to cause around 10, 000 stings each year on the east coast of Australia alone [16]. While no definite fatalities have been attributed to *Physalia *in Australia, three deaths have been attributed to *Physalia physalis *on the Atlantic coast of the USA [16]. The most dangerous jellyfish in the world is the 'Box jellyfish', *Chironex fleckeri*, which, although its range has yet to be accurately defined, has been identified off northern Australia from latitude 27° south to approximately 18° north along the coast of Vietnam [16,17]. *C. fleckeri *has been responsible for fatalities in Nuigini, The Philippines and Malaysia and for around 70 deaths in tropical Australian waters [15,16]. *C. fleckeri *is an in-shore or littoral species and stings usually occur to victims who are wading or swimming in shallow water [15,16]. Although scientists conduct research that may directly or indirectly involve potential contact with this jellyfish [14,18], most *C. fleckeri *stings, however, are not life-threatening and severity of injury is related to the size of the jellyfish and the extent of tentacle contact [13,16,60]. Fatality is usually due to cardiopulmonary arrest within minutes of the sting occurring [15,16].

There are many other jellyfish species which can cause painful stings to humans including: *Chiropsalmus quadrigatus*, a species of 'box jellyfish' which is a major cause of stings in the Indo-Pacific and Brazil, and has been responsible for at least one death in Mexico; 'Irukandji', *Carukia barnesi*, whose victims may present with a variety of symptoms such as abdominal cramps and vomiting and which has been reported in Australia, Nuigini, Hawaii, Japan and China; and 'The Little Mauve Stinger', *Pelagia noctiluca*, and the 'Hair Jelly', *Cyanea capillata*, which have wide distributions in the world's oceans and which, on contact, can cause immediate, local pain [16].

Other marine animals responsible for sting or bite injuries, if disturbed or handled, include: scorpion fish or stonefish [21,36], catfish [20], moray eels [22], blue-ringed octopus [19], sea snakes [48], sponges [10] and cone shells [60], however these animals are routinely handled by marine researchers and severe effects from sting or bite injuries from these animals are rare [55].

Of more concern to freshwater researchers are snakes at the water's edge as they are responsible for at least 5 million bites, with 2.5 million envenomations and 100, 000 deaths each year across Africa, Asia, and the Americas [55]. In comparison, only 151 cases of snake bite were identified in Western Australia between 1994 and 2004, with the mean age of patients being 6.6 years, 55% were male, and most were bitten on the lower limb (72%) [54]. Furthermore, in Queensland, Australia, only 211 (3%) of all farming injuries between 1998 and 2005 were due to snake bite [57].

Generally, in Australia, the home is the most common location for bites to occur with animal bites and stings being attributed to bats, cats, rats, spiders, horses, snakes, possums, rabbits and platypus. However, dogs are responsible for the majority of bites, injuring around 100, 000 people each year [55,58,59]. Treatment for pain relief from stings or bites is relatively common in the literature [15,16,54,56,61], however, despite the relatively low risk, it is advisable that if an aquatic researcher must enter the water, vigilance is required when handling animals and protective clothing such as wetsuits and gloves should be worn, particularly in tropical waters.

### Scuba diving

SCUBA diving is a popular activity which can occasionally result in severe injury or death (Table 2). The number of diving-related fatalities reported in North America from 1970 to 2004 ranged from 80 to 120 annually [62]. There were 24 fatalities in Western Australia between 1992 and 2005 [63] and 40 fatalities in Okinawa, Japan, between 1982 and 2007 [64,65]. Worldwide, 138 diving-related deaths were reported in 2006 [66]. The number of divers who are treated with recompression therapy for severe dive-related complications or survive diving injuries without reporting them, however, far exceeds these figures [64,67,68]. Although a significant number of SCUBA-related injuries and deaths occur to novices or uncertified divers [65,69], the potential for death, injury or impact on long term health must be considered by the research diver because of the increased exposure to relevant hazards.

**Table 2:** SCUBA diving-related occupational health issues in marine and freshwater research

Title of article	Year^a^	Authors
Analyses of variables underlying US Navy diving accidents	1985	Blood & Hoiberg [70]

Neurological long term consequences of deep diving	1991	Todnem et al. [71]

Carbon monoxide poisoning in a diver	1992	Allen [72]

Diving and hyperbaric ophthalmology	1995	Butler [73]

Diving and marine medicine review part II: diving diseases	1999	Spira [64]

Patterns of drowning in Australia, 1992-1997	1999	Mackie [74]

Lung function over the first 3 years of a professional diving career	2000	Skogstad et al. [75]

The "bends" and neurogenic bladder dysfunction	2001	Elliott et al. [76]

Neurologic complications of scuba diving	2001	Newton [67]

Diving medicine: contemporary topics and their controversies	2001	Strauss & Borer [77]

Arterial gas embolism and decompression sickness	2002	Neuman [78]

Decompression illness in divers: A review of the literature	2002	Barratt et al. [79]

Incidence and risk factors for symptoms of decompression sickness among male and female dive masters and instructors--a retrospective cohort study	2003	Hagberg & Őrnhagen [68]

British Thoracic Society guidelines on respiratory aspects of fitness for diving	2003	Godden et al. [80]

Differential diagnostic problems of decompression sickness--examples from specialist physicians' practices in diving medicine	2003	Petri & Andrić [81]

Diving medicine	2006	Benton & Glover [82]

Risk factors for dysbaric osteonecrosis	2006	Miyanashi et al. [83]

Risk factors for dive injury: a survey study	2007	Beckett & Kordick [69]

Pressure related incidence rates in scientific diving	2007	Dardeau & McDonald [1]

Dysbaric osteonecrosis in experienced dive masters and instructors	2007	Cimsit et al. [84]

Reduced health-related quality of life in former North Sea divers is associated with decompression sickness	2007	Irgens et al. [85]

Health status of professional divers and offshore oil industry workers	2007	Ross et al. [86]

The nontechnical causes of diving accidents: Can U.S. Navy divers learn from other industries?	2007	O'Connor [87]

Neurologic injuries from scuba diving	2008	Hawes & Massey [62]

Annual Diving Report: 2008 Edition	2008	DAN [66]

Dysbaric osteonecrosis screening in Turkish Navy divers	2008	Uzun et al. [88]

Physical training of combat diving candidates: implications for the prevention of musculoskeletal injuries	2008	Pelham et al. [89]

Western Australian recreational scuba diving fatalities, 1992 to 2005	2009	Buzzacott et al. [63]

Scuba-diving related deaths in Okinawa, Japan, from 1982 to 2007	2009	Ihama et al. [65]

Diving medicine: a review of current evidence	2009	Lynch & Bove [90]

^a ^Year of publication

The work of a research diver may range from simple observations to use of sophisticated technology, however risks may include cold and arduous dives, task loading, time-at-depth limitations and working with heavy machinery. While reporting of diving-related occupational incidents may be incomplete, the US Occupational Safety and Health Administration (OSHA) compared diving-related occupational incidents in the periods 1965-1981 and 1998-2005, and despite increased occupational activity during the latter period, an incident rate of around 1% of scientific divers per year was estimated for both periods [1]. Additionally, a review of US military diving between 1968 and 1981 reported 1174 adverse events from 706, 259 dives (0.2%) [70]. However, these figures relate to diving practices of more than 30 years ago and military diving procedures may differ from civilian recreational practices.

Divers tend to have larger lung volumes than standard reference populations, and perhaps due to repetitive breath holding and resistive breathing during diving activities, one long term-effect on occupational divers is a reduction in vital capacity, indicating changes in airway function [75]. Furthermore, limited data from longitudinal studies of commercial divers suggest that their lung volumes decline at a faster than expected rate [80].

After drowning, which is the cause of 60% of all US diving fatalities and 56 diving fatalities in Australia between 1992 and 1997, pulmonary-related illnesses are the most common cause of death in divers, accounting for nearly a quarter of fatalities each year [62,64,65,67,74]. Arterial Gas Embolism (AGE) is a pulmonary barotrauma which arises during ascent from gas expansion which exceeds the lungs' elastic ability, resulting in alveoli rupture. Bubbles of gas move from the alveoli into the bloodstream, lodging in arterioles and capillaries, particularly in the brain, where they impede blood flow, compromise the blood-brain barrier, and result in respiratory distress, stroke-like events or death [62,64,90]. Other potential barotraumas include ear, sinus and dental which are most likely to occur on descent and may result in acute pain due to compression of gas-filled cavities in the diver's head [64,90]. Although not fatal, these barotraumas can be extremely painful.

Decompression sickness (DCS), or the "bends', is a well known risk of SCUBA diving, although its reported incidence in the diving community is generally low (≈ 1%) [64,67,76,81]. Similar to AGE, DCS is caused by nitrogen gas coming out of solution and forming bubbles in blood and tissues if ambient pressure is released too quickly [81,90]. These bubbles may accumulate in skin capillaries and around tendons, ligaments and muscles typically causing joint pain. Almost all symptoms usually occur within 6-12 hr of surfacing; however, symptoms vary significantly. Furthermore, if a diver is flying soon after diving, the risk of DCS is increased since, at high altitudes, reduced cabin pressure may result in gas bubble expansion in the capillaries. Consequently, an interval of 12-24 hr between the last dive and flying is recommended [64,82]. Severe DCS, which is more commonly experienced by recreational divers, can be more life threatening if bubbles restrict blood flow to the lungs or block blood flow in nerve tissue, possibly causing symptoms such as numbness and paralysis [64,76,81]. Since the signs and symptoms of severe DCS and AGE may overlap in the same victim, these two conditions may be difficult to differentiate [78]. Furthermore, both conditions may result in ophthalmic problems such as ocular barotraumas, optic neuropathy and visual field defects [73]. Nevertheless, both conditions may be resolved at the surface with the immediate breathing of pure oxygen which tends to eliminate the nitrogen bubbles from tissue [62,77]. Occupational divers, although more aware of SCUBA diving safety, are more likely to experience decompression illnesses and may frequently self diagnose and treat unreported symptoms with oxygen that is readily available on diving expeditions [68,79].

Prolonged exposure to compressed air and inadequate recompression can result in Dysbaric Osteonecrosis (DON) which occurs when nitrogen gas expands in bone marrow tissue. While one Turkish study concluded that the risk of DON is very low for military divers who strictly obey the decompression rules and who undergo periodic medical examination [88], another Turkish study of volunteer divers who had performed at least 500 dives, working as a dive master or instructor, but who had never performed industrial or commercial dives, detected DON lesions in 25% of the divers [84]. Furthermore, a Japanese study of occupational divers detected evidence of DON in 55% of sample divers, with a higher incidence of DON lesions in divers who usually dived to depths of greater than 30 m compared with divers who dived to shallower depths [83]. Since the basic causes of this illness, however, are not fully understood, DON remains a significant occupational health hazard.

Occupational divers are more likely than recreational divers to incorporate mixed gas, re-breather and deep-diving techniques, and these practices are becoming more commonly used by marine researchers. Mixed gas and re-breather methods enhance the amount of oxygen in the gas mixture which results in less deposition of nitrogen in body tissue and reportedly reduces fatigue during diving activities [77]. While these techniques may extend dive time required for deep diving work, oxygen toxicity and sufficient decompression during ascent must be considered. Furthermore, the increased hyperbaric pressure experienced by commercial saturation divers appears to have a long-term impact on the nervous system with neurological symptoms being significantly correlated with exposure to deep diving and prevalence of DCS [62,71,85,86].

While diving equipment has improved and contributed to diver safety, there is still the potential for equipment failure, perhaps due to poor maintenance, or misuse. Faulty air compressors may result in contaminated air supply, power inflators on buoyancy compensators can become locked open and result in uncontrolled ascent, and dive computers may fail [64,72,77]. Although computer-assisted diving has become commonplace, Spira (1999) outlined a Royal Navy study which revealed that 84% of the subjects had dived outside the limits of their computers and 8% of accident cases recorded were by divers who used neither decompression tables nor computer. Occupational diving is a potentially high risk activity and is also physically demanding [89]. However, with appropriate training, extensive supervision and preparation, effective teamwork and situation awareness in occupational circumstances, the potential for accidents to occur can be minimised [87]. While data certainly exists for health and safety incidents relating to occupational diving, few articles describing specific OH&S diving issues for marine researchers were evident.

### Exposure to toxins and carcinogens

Illness and death following consumption of molluscs, gastropods, crustaceans and fish have long been attributed to 'natural' toxins from toxic microbes bioaccumulating via the food chain [91]. However, microbial contamination of seafood by bacteria, viruses and protists, related directly and indirectly to anthropogenic activity, is increasingly posing a risk to the safety of seafood supply [6]. Additionally, contamination from industrial and urban sources such as sewage, wastewater, various fungicides and insecticides, and sediment leachates is also of concern to public health [92]. Recreational and occupational users of the marine and freshwater environments are clearly exposed to the contamination and the growing number of recorded incidences of gastrointestinal, respiratory, dermatological, and ear, nose, and throat infections is of particular concern considering the estimated public health costs and loss of income to aquatic researchers worldwide (Table 3) [4,6,93].

**Table 3:** Toxin and carcinogen-related occupational health issues in marine and freshwater research

Title of Article	Year ^a^	Authors
Toxic marine and freshwater algae: an occupational hazard?	1991	Baxter [4]

Marine toxins	2000	Whittle & Gallacher [91]

Marine swimming-related illness: implications for monitoring and environmental policy	2001	Henrickson et al. [93]

Cancer incidence among marine engineers, a population-based study (Iceland)	2003	Rafnsson & Sulem [94]

Cancer risk to naval divers questioned	2003	Amitai et al. [95]

Listing occupational carcinogens	2004	Siemiatycki et al. [96]

Factors influencing organotin distribution in different marine environmental compartments, and their potential health risk	2006	Lee et al. [92]

Oceans and human health: Emerging public health risks in the marine environment	2006	Fleming et al. [6]

A survey of diving behaviour and accidental water ingestion among Dutch occupational and sport divers to assess the risk of infection with waterborne pathogenic microorganisms	2006	Schijven & de Roda Husmen [5]

Cancer risks in naval divers with multiple exposures to carcinogens	2009	Richter et al. [3]

^a ^Year of publication

Schijven and de Roda Husman (2006) suggest that divers may run a higher risk of infection with waterborne pathogens than bathers because of more frequent and intense contact with water that may not comply with microbiologic water quality standards for bathing water. With increased exposure to polluted waters, occupational divers' skin lesions and skin infections provide greater opportunities for microorganisms and toxic chemicals to penetrate. Also, working divers experience an elevated incidence of gastroenteritis and a significantly higher frequency of *Pseudomonas *and *Aeromonas *in ears and respiratory surfaces [5]. Occupational divers have been found to swallow more water per dive than sports divers, less water when wearing a full face mask instead of an ordinary diving mask and even less when wearing a diving helmet [5]. Consequently, a full face mask or a diving helmet sealed to a special drysuit is recommended when conducting sampling activities in faecally or chemically contaminated water.

Occupational exposure to chemicals may be related to a high incidence of cancers amongst some marine workers [94,97]. Raffnsson and Sulem (2003) studied a cohort of over 6000 marine engineers over a 40-year period to 1998 and concluded that an increased risk of mesothelioma was possibly attributable to previous asbestos exposure, and a high incidence of stomach, lung and bladder cancers may have been related to exposure to oils and petroleum products. Risk of hemotolymphopoietic, central nervous system, gastrointestinal and skin cancers was also increased in naval commando divers with prolonged underwater exposure to a waterway severely polluted by industrial, ship and agricultural effluents [3]. Furthermore, melanoma incidence in the study cohort was notably higher in those divers using defective and torn skin suits [95].

Unfortunately, published research conducted on specific health risks to environmental workers due to their occupational exposure to toxins and carcinogens is limited. Many occupational exposures are also found in the general environment and, among many substances now in the industrial environment, there has been no quantitative risk assessment using human data [96].

### Boating

One of the fundamental tools of an aquatic researcher is a boat. Sea sickness; hazardous conditions; an error of judgement; unsafe work practices; and failure to wear a personal flotation device (PFD) are the main contributing factors to injury and death attributable to boating accidents. In Australia, deaths of commercial fishers are the most common cause of work-related maritime fatality. Death rates have decreased from 143 fatalities in 1982-1984 to 74 in 1992-1998, mainly through drowning while not wearing a PFD [98]. Additionally, in Great Britain, the fatal accident rate for fishers was higher than for all other workers between 1976 and 1995 [99]. Again, however, there is no substantial data available on specific work-related boating injuries to aquatic researchers.

## Conclusion

Overall, this review suggests that marine and freshwater scientists are potentially exposed to a wide variety of occupational hazards. Depending on the focus of their research, risks may include animal attacks, physiological stresses, exposure to toxins and carcinogens, and dangerous environmental conditions. Considering that many of these hazards are also applicable to recreational users, very few studies have specifically examined potential hazards from an occupational perspective. Marine and freshwater research is a potentially risky occupation, and therefore the possibility of death, injury and long-term health impacts needs to be seriously considered. However, the consequences of increased exposure to specific hazards that marine and freshwater scientists may experience due to their activities, whether they be in the laboratory or the field, have received little attention, and as such, the magnitude of the problem is relatively unknown. While this article provides, what we believe to be, the most comprehensive and up to date review of literature describing the hazards faced in marine and freshwater research, it is possible that some references and reports may have been missed during the literature search, especially if they were published in languages other than English. Nevertheless, further studies are needed to elucidate the significance of the hazards discussed and their impact on particular research activities. In the meantime, scientists must remain aware of the potential hazards and develop appropriate risk assessment procedures when conducting research in the marine or freshwater environment.

## Competing interests

The authors declare that they have no competing interests.

## Authors' contributions

GC conducted the research and drafted the manuscript. DRS conceived the idea and assisted with the research and drafting of the manuscript. WG assisted the research and drafting of the manuscript. All authors read and approved the final manuscript.
